# Transport and Electrochemical Interface Properties of Ionomers in Low-Pt Loading Catalyst Layers: Effect of Ionomer Equivalent Weight and Relative Humidity

**DOI:** 10.3390/molecules25153387

**Published:** 2020-07-26

**Authors:** Sushmit Poojary, Muhammad Naoshad Islam, Udit N. Shrivastava, Edward P. L. Roberts, Kunal Karan

**Affiliations:** Department of Chemical and Petroleum Engineering, University of Calgary, Calgary, AB T2N 1N4, Canada; sushmit.poojary@ucalgary.ca (S.P.); muhammadnaoshad.isla@ucalgary.ca (M.N.I.); udit.shrivastava@ucalgary.ca (U.N.S.); edward.roberts@ucalgary.ca (E.P.L.R.)

**Keywords:** catalyst layer, polymer electrolyte fuel cell, oxygen transport resistance, oxygen reduction reaction kinetics, platinum ionomer interface, ionomer thin film

## Abstract

Catalyst layer (CL) ionomers control several transport and interfacial phenomena including long-range transport of protons, local transport of oxygen to Pt catalyst, effective utilization of Pt catalyst, electrochemical reaction kinetics and double-layer capacitance. In this work, the variation of these properties, as a function of humidity, for CLs made with two ionomers differing in side-chain length and equivalent weight, Nafion-1100 and Aquivion-825, was investigated. This is the first study to examine humidity-dependent oxygen reduction reaction (ORR) kinetics in-situ for CLs with different ionomers. A significant finding is the observation of higher ORR kinetic activity (A/cm^2^_Pt_) for the Aquivion-825 CL than for the Nafion-1100 CL. This is attributed to differences in the interfacial protonic concentrations at Pt/ionomer interface in the two CLs. The differences in Pt/ionomer interface is also noted in a higher local oxygen transport resistance for Aquivion-825 CLs compared to Nafion-1100 CLs, consistent with stronger interaction between ionomer and Pt for ionomer with more acid groups. Similar dependency on Pt utilization (ratio of electrochemically active area at any relative humidity (RH) to that at 100% RH) as a function of RH is observed for the two CLs. As expected, strong influence of humidity on proton conduction is observed. Amongst the two, the CL with high equivalent weight ionomer (Nafion-1100) exhibits higher conduction.

## 1. Introduction

**Figure 1:**
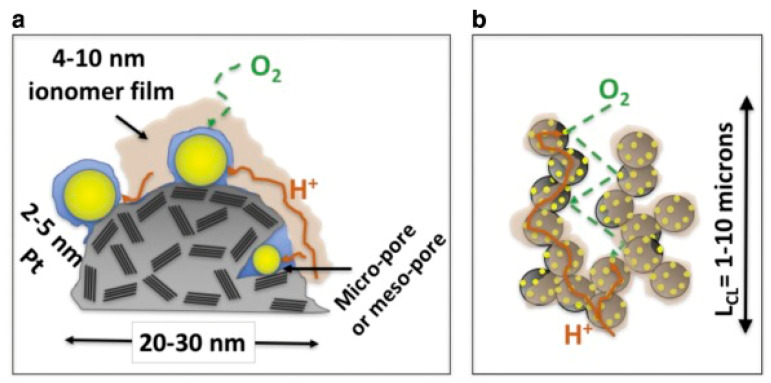
Depiction of microstructure of and key processes in a polymer electrolyte fuel cell (PEFC) catalyst layer. (**a**) Local transport of oxygen and protons to the surface of Pt catalyst particles (yellow) on the surface and in the micropores of carbon support coated with ionomer thin film (**b**) Long-range transport of proton and oxygen transport through the thickness of the catalyst layer. Reproduced with permission from Karan [3], Current Opinions in Electrochemistry; published by Elsevier, 2017.

Ion-containing polymer or ionomer is a key material in polymer electrolyte fuel cells (PEFCs). Until a decade ago, the focus of studies on ionomeric materials and their fuel cell properties were largely limited to their application as the polymer electrolyte membrane separating the anode and the cathode. However, ionomer is also one of the critical material constituents of the cathode and the anode catalyst layers. Catalyst layers of the polymer electrolyte fuel cells are complex, nanoporous, nanocomposite of ionomer, and Pt/C catalyst with co-continuous phases [1,2,3]; see Figure 1 [3]. In a typical Pt/C-based catalyst layer, a 4–10 nm thin film of acid ionomer covers the aggregates of Pt/C catalyst [4]. The catalyst layer ionomer is often described as a binder [5], which does not capture the multiple crucial functions it serves: (i) as an ion-conducting material phase that ensures transport of protons over 10–20 micron thick catalyst layers, (ii) as an acidic medium that together with Pt catalyst forms the electrochemically active interface where the oxygen reduction reaction (cathode) or hydrogen oxidation reactions (anode) occur, and (iii) as a material phase that controls the transport of reactants (O_2_ or H_2_) and products to/from the active Pt sites.

The ionomer in a catalyst layer is essential for facilitating proton transport to achieve high in-operando electrochemical surface area (ECSA) utilization [6]. Pt catalysts not in direct contact with ionomer (see Figure 1a) may not be accessible to protons under low relative humidity (RH) conditions. Recently, it has been identified that the chemical structure of ionomer dictates the chemical environment of the electrochemically active interface including poisoning by sulfonic group and ultimately affecting the oxygen reduction reaction (ORR) activity [7].

Oxygen transport through the ionomer thin film covering the catalyst is known to be a limiting factor for low-Pt loading catalysts [8,9,10,11]. It is now well established that confinement effects and interactions with substrates (e.g., Pt/C) strongly influence the structure and properties of the ionomer such that it differs significantly from that of the free-standing membrane [12,13,14,15,16,17,18,19,20]. The proton conduction, the nature of the electrochemical interface, and the local transport of reactants in the catalyst layer ionomer all depend on its hydration state (water content). In addition to bulk water content, the interfacial water content also becomes important for various transport processes and electrochemical phenomena in catalyst layer ionomers. Both bulk and interfacial water content depend on the relative humidity (RH). Exposure to humid environment stimulates the evolution of hydrophilic domains in the bulk ionomer and water sorption at the Pt-ionomer interface [15]. Hydrophilic domains are necessary for proton transport [21] and also assist in oxygen permeation through the thin film [11,22]. Hydration can lead to reorganization of surface, bulk, and buried interfacial structure of ionomers [23,24]. Although discussed sparingly in the literature, it can be expected that interfacial water will influence (a) the extent of acid dissociation, i.e., local pH, and thereby the electrochemical reaction kinetics, (b) the conduction of protons on the catalyst surface, and (c) the transport resistance of oxygen to the Pt catalyst. Moreover, increase in RH enhances the accessibility of Pt catalyst residing in the internal pores of the support and thus affects ECSA [25]. The chemical structure of ionomer and the chemical nature of substrate surface affect the size of the hydrophilic domains in the bulk and at the interface [14,26].

A handful of studies in the literature reports the effect of ionomer type on fuel cell performance [5,27,28]. Even fewer studies discuss the ionomer-dependent relevant catalyst layer electrochemical properties such as ECSA, double-layer capacitance, kinetics, and the local oxygen transport resistance [29,30,31,32] and no single study discusses all of these properties for the same catalyst layer. Incorporation of higher ion exchange capacity (IEC) ionomers improve ECSA and specific activity [29] but at the expense of higher local oxygen transport resistance [31]. However, only a few studies have examined the effect of RH on the aforementioned characteristics—ECSA, ORR kinetics, double-layer capacitance, oxygen transport resistance—of the catalyst layer. The literature lacks a systematic account of the combined effect of ionomer structure and RH on the catalyst layer electrochemical properties. In addition, the discussion of the catalyst layer electrochemistry in the context of Pt/ionomer interface is absent. Overall, humidity-dependent probing of catalyst layer properties can provide significant insight into how ionomer molecular structure influence both transport properties and Pt/ionomer interfacial characteristics.

**Scheme 1:**
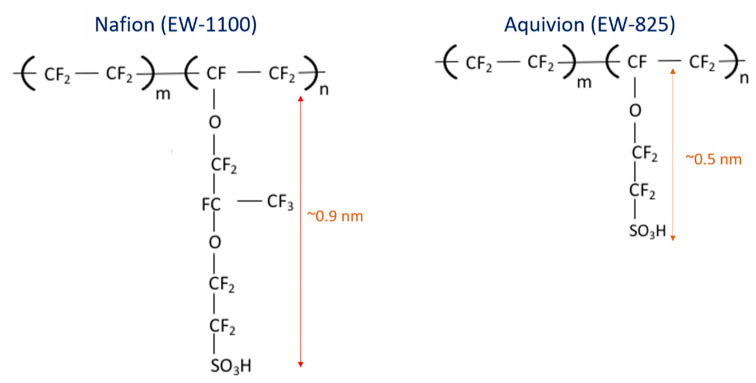
Chemical structure of Nafion and Aquivion ionomers.

Here, the RH-dependent bulk and interfacial properties of catalyst layer ionomer are reported for long side chain (LSC) Nafion ionomer with an equivalent weight (EW) of ca. 1100 (Naf-1100) and short side chain (SSC) Aquivion ionomer, EW ca. 825 (Aq-825) as a function of relative humidity. Scheme 1 presents the molecular structure of both ionomers. The method of catalyst layer fabrication and details of experimental measurements are provided in the Materials and Method section.

## 2. Results

The RH-dependent electrochemical and transport properties for low-Pt loading catalyst layers (nominal Pt loading of 0.04 mg/cm^−2^) prepared with 10 wt% Pt/C catalyst and ionomer:carbon ratio of 0.8 for the two different ionomers are reported. Electrochemical properties studied include the double-layer capacitance, electrochemically active surface area, and ORR kinetics, while the transport properties studied are the proton conduction and oxygen transport resistance.

**Figure 2:**
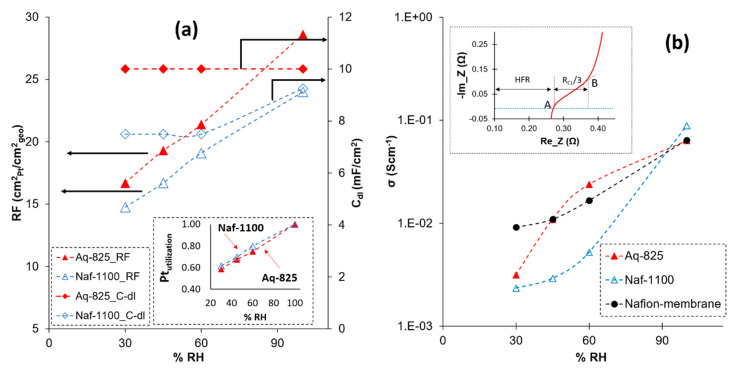
Comparison among relative humidity (RH)-dependent properties of Naf-1100 and Aq-825 derived from cyclic voltammetry (CV) and electrochemical impedance spectra (EIS) in H_2_/N_2_ at 70 °C and 100% RH (**a**) roughness factor and double-layer capacitance (**b**) protonic conductivity.

Double-layer capacitance, electrochemically active surface area, and protonic conductivity: The electrochemically active surface area obtained from H-adsorption peak integration from cyclic voltammetry (CV) scan was normalized with respect to the electrode area and denoted as the roughness factor (RF). Figure 2a compares the RH-dependent RF and double-layer capacitance (*C_dl_*) for the catalyst layers Aq-825 and Naf-1100. RF for both ionomers increases with RH, similar to that reported in other studies [8,25], whereas *C_dl_* almost remains constant for Aq-825 but increases with RH for Naf-1100. *C_dl_* mainly arises from the interfacial charges at the ionomer/carbon and ionomer/Pt interface. Differences in the catalyst layer microstructure or interfacial characteristics can result in the differences in *C_dl_* for two catalyst layers. If the ionomer coverage on the carbon for the two catalyst layers is different, it would result in different *C_dl_*. If the charge concentration at the ionomer/carbon and ionomer/Pt interface is different, it could also result in different *C_dl_*. The higher magnitude of *C_dl_* for Aq-825 catalyst layer than Naf-1100 catalyst layer points toward either a higher coverage of ionomer in Aq-825 catalyst layer or high interfacial charge concentration than that in Naf-1100 catalyst layer. Although the ionomer to carbon ratio was kept the same for the two catalyst layers and similar coverages are expected, we do not have direct evidence of the microstructural similarity. Thus, the origin of differences in magnitude of *C_dl_* as well as its RH-dependence remains unresolved. Only one previous study that has reported *C_dl_* of catalyst layers with different ionomers [31] could be found. In that study, for catalyst layers made with Nafion and Aquivion ionomers with similar ionomer loading (30 wt%), the *C_dl_* was reported to be 19 mF cm^2^. However, it was also reported that a catalyst layer with lower ionomer content (10 wt% Aquivion ionomer) had higher *C_dl_* than that for high ionomer content catalyst layer, which is counterintuitive. To the best of our knowledge, no studies have reported a comparison of RH-dependent *C_dl_* of CLs with different ionomers. Thus, the differences in RH-dependent *C_dl_* observed for two ionomers in this study cannot be cross-checked with results from other studies. Moreover, it is not so straightforward to delineate the effect of RH on double-layer capacitance. The double-layer capacitance is known to be pH-dependent and complicated by the contributions from charges in the Helmholtz and diffuse double layer, even in simpler liquid electrolyte/bare metal electrode systems [33]. Double-layer capacitance in such systems usually increases with decreasing pH. However, the system studied here has a solid electrolyte (ionomer), wherein the water content is controlled by RH. The interfacial water will control the interfacial pH, while the bulk water (in the ionomer phase) will control the connectivity of the proton-conducting water channel. Thus, while the interfacial water controls the charge distribution across the solid/polymer interface, the bulk water controls the accessibility of the protons to the interface during potential scanning. Interestingly, for Aq-825, in dry conditions, the CL double-layer capacitance was 1.33 times greater than that of the Naf-1100 CL, which is equal to the ratio of their ion exchange capacities (IECs). At a given RH, the RF for Aq-825 catalyst layer is greater than that of the Naf-1100 catalyst layer. The electrochemical surface area (ECSA) at 100% RH for Aq-825 catalyst layer is 78 m^2^/g_pt_, while that for Naf-1100 catalyst layer is 56 m^2^/g_pt_, indicating higher Pt accessibility in Aq-825 catalyst layer. A previous study also reported a higher Pt utilization for catalyst layer prepared with high IEC (EW 980) Aquivion ionomer than that prepared from low IEC (EW 1100) Nafion ionomer [29]. Both higher IEC and, possibly, higher coverage of Pt/C by ionomer in Aq-825 catalyst layer than in Naf-1100 catalyst layer must contribute to the higher *C_dl_* as well as RF of Aq-825 catalyst layer. The RH-dependent Pt utilization was calculated by dividing ECSA at any RH by the ECSA at 100%RH. The Pt utilization (see inset of Figure 2a) of the two catalyst layers at any given RH was very similar, indicating that the type of ionomer does not affect the RH-dependent Pt utilization. For 10 wt% Pt/Vulcan catalyst, Padgett et al. [25] reported that Pt utilization was dependent on RH. From tomography performed on 10 wt% Pt particles, they found that Pt particles located in the internal pores contributed about 20–30% to the total surface area. However, they did not report the double-layer capacitance, and as such a correlation between ECSA accessibility and double-layer capacitance is not available from their study. The observed increase in RF with RH could be due to the increased accessibility of protons to the Pt in the internal pores.

Figure 2b shows the variation of the protonic conductivity of Aq-825 and Naf-1100 catalyst layers as a function of humidity. For comparison, the protonic conductivity of Nafion membrane has also been included. The protonic resistances for the catalyst layers and the membrane were determined from analyses of the electrochemical impedance spectra (EIS) at each RH. The inset of Figure 2b shows an example of the impedance spectra at 30% RH in H_2_/N_2_. Similar impedance spectra were used at each RH to estimate the high-frequency resistance or HFR (marked by point A in inset of Figure 2b) and the catalyst layer protonic resistance R_CL_. The high-frequency resistance is the sum of the electrical and membrane protonic resistances, and Equation (1) below was used to calculate the membrane protonic conductivity.

(1) $$\sigma_{mem}=\frac{t_{mem}}{HFR-R_{e}}$$

where $\sigma_{mem}$ is the membrane conductivity, *R_e_* is area-specific electrical resistance (10 mΩ cm^2^) determined from an ex-situ resistance measurement, and *t_mem_* is the thickness of the membrane. In a typical EIS of a fuel cell catalyst layer, the point along the 45° line at which spectra transition to a completely capacitive behavior, for example, point B in the inset, the real component of spectra at this point is summation of HFR and a third of *R_CL_* [34]. Using Equation (2) (see below), the catalyst layer conductivity is obtained [35].

(2) $$\sigma_{CL}=\frac{t_{CL}}{R_{CL}\varepsilon^{n}}$$

where $\sigma_{CL}$ is catalyst layer conductivity, *ε* is ionomer volume fraction, the Bruggeman exponent *n* is 1.5 [34], and $t_{CL}$ is thickness of the catalyst layer.

Expectedly, protonic conductivity of catalyst layer and membrane are strong functions of RH. At 30% RH, both Aq-825 and Naf-1100 catalyst layer conductivities are almost an order of magnitude smaller than the Nafion membrane conductivity, and at any given RH, Naf-1100 catalyst layer conductivity is smaller than the Aq-825 conductivity. In a catalyst layer, ionomer exists in thin film form (depiction shown in Figure 1) and it is known that at comparable RH, ionomer thin film exhibits much lower protonic conductivity than the bulk membrane [13]. The IEC of Aq-825 ionomer (IEC ~1.2) is higher than that for Naf-1100 ionomer (IEC ~0.9). The difference in conductivity can be attributed to the intrinsic effect of difference in acid content of the ionomers and to the extrinsic effect of how ionomer is spatially distributed in the catalyst layer. The former effect is well known for bulk membranes, while the latter effect (differences in microstructure) is complicated. For example, lower coverage of ionomer would imply thicker ionomer films that have higher conductivity but the connectivity and tortuosity may also be higher.

### 2.1. Oxygen Reduction Reaction (ORR) Kinetics

Little is understood about the influence of ionomer EW and side chain on the ORR kinetics. Ionomer EW or IEC is a measure of its acidic strength. From early studies of ORR on Pt electrodes in liquid electrolyte, Damjanovic and Brusic proposed the following kinetic expression highlighting the dependency of ORR kinetic current ($i_{ORR}$) on proton activity/concentration [36]:

(3) $$i_{ORR}=k\;P_{O_{2}}{}^{n}\;{\left[H^{+}\right]}^{m}\;exp\left(-\frac{\alpha \;F}{RT}\eta_{ORR}\right)$$

where *k* is the electrochemical rate constant akin to exchange current density, *n* and *m* are reaction order stated to be 1 and 1.5 [36], *α* is transfer coefficient, $p_{O_{2}}$ is oxygen partial pressure, $\eta_{ORR}$ is ORR overpotential, *F* is Faraday constant, *R* is universal gas constant, and *T* is temperature. It must be noted that in the original work [33], the formal potential rather than overpotential was used.

**Figure 3:**
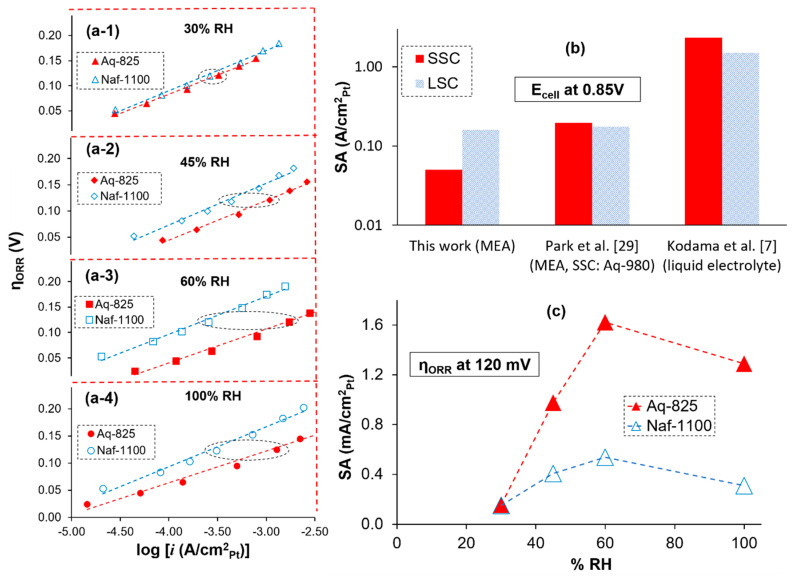
Comparison among kinetic properties of Naf-1100 and Aq-825 in H_2_/air (**a**) activation overpotential against current density in Tafel region at different RHs (**b**) specific activity at 0.85 V from this work, park et al. [29], Kodama et al. [7] in liquid electrolyte (**c**) specific activity at activation overpotential of 120 mV and at different RHs.

The interfacial proton concentration in the catalyst layer, i.e., proton concentration at the Pt/ionomer interface, would depend on the abundance of sulfonic acid groups at or near the Pt/ionomer interface as well as the interfacial water content, both of which are not directly accessible in fuel cell experiments. From our neutron reflectometry (NR) study of different ionomers on planar Pt, it is known that the amounts of water present at the Pt/ionomer interface varies with RH and at 100% RH the water content correlates to the ionomer side-chain length [12,14]. However, the abundance of sulfonic group is not known. CO desorption electrochemistry applied to estimate Pt-sulfonic group interactions could offer insight into this but was not applied in the current study [37]. Regardless, it can be expected that the proton concentration at the Pt/ionomer interface would vary with RH and could be different for different ionomers. Accordingly, proton concentration-dependent ORR kinetics (Equation (3)) can be expected to occur. A comparison of RH-dependent ORR kinetic behavior of Aq-825 and of Naf-1100 catalyst layers is presented in Figure 3. By neglecting oxygen transport resistance and hydrogen oxidation reaction overpotential, the *η_ORR_* was estimated using Equation (4) below and plotted against the log of specific current density (*i_s_*, A cm^−2^_Pt_), see Figure 3a (1–4), clearly following the Tafel behavior.

(4) $$\eta_{ORR}=OCV-E_{cell}-i\;\left(HFR+R_{CL}/3\right)$$

where OCV is open-circuit voltage, *i* is current density, and *E_cell_* is cell voltage.

For most electrochemical reactions, two kinetic parameters are often considered—the Tafel slope, which is related to the transfer coefficient (*α*), and the exchange current density. From Figure 3a, it can be noted that the slopes of the plot, which is the Tafel slope, are similar for both catalyst layers at each RH. The Tafel slopes were found to be 66–70 mV/decade. It is also obvious from the data in Figure 3a that at each RH, ORR current in the Aq-825 catalyst layer is superior to that in the Naf-1100 catalyst layer. Since Tafel slope (or α) are similar, considering the Damjanovic and Brusic formulation of ORR kinetics [36], it can be deduced that higher current density (expressed on a per cm^2^ of Pt basis) for Aq-825 catalyst layer compared to that for Naf-1100 catalyst layer would be a result of higher interfacial concentration of protons at Pt/ionomer interface for Aq-825 catalyst layer. The higher IEC of Aq-825 and its shorter side chain can be expected to create a higher abundance of sulfonic groups at the interface and, thereby, in a higher interfacial proton concentration. To the best of our knowledge, there has been no prior study comparing RH-dependent ORR kinetics for catalyst layers with different ionomers.

In literature, the kinetic performance of a catalyst layer is generally evaluated by defining specific activity or mass activity at a voltage in kinetic or Tafel region of the polarization curve. In Figure 3b, the specific current density, i.e., specific activities (SA) for ORR at 0.85 V for catalyst layers made with short and long side chain (SSC and LSC) ionomers in this work are compared along with results from other studies for SA determined in membrane electrode assembly (MEA) [29] and in liquid electrolyte [7]. In MEAs, regardless of the type of ionomer used in fabricating the catalyst layer, SA at 0.85 V in MEA can be as much as ten times lower than that in liquid electrolyte (rotating disc electrode or RDE setup) [7]. In the present work, SA at 0.85 V in 100% RH for Aq-825 catalyst layer is lower than that for Naf-1100 catalyst layer. Since OCV for the Aq-825 catalyst layer at 100% RH is 40 mV lower than the Naf-1100 catalyst layer, as per Equation (4), *η_ORR_* for both cells are different at same *E_cell_* and, hence, comparison of SA at same voltage may not be valid. Therefore, SA in Figure 3c is compared at a similar *η_ORR_* = 120 mV marked by the dotted ellipses in Figure 3a. Except at 30% RH, at each RH, SA for Aq-825 catalyst layer is 2–5 times greater than Naf-1100 catalyst layer. For both catalyst layers, SA increased with RH. From 30% RH to 60% RH, SA for Aq-825 SA catalyst layer increased ten folds whereas for Naf-1100 SA increased by three times. Then, for both catalyst layers between 60% and 100%, RH SA decreases—trend is similar to a previous study [29].

### 2.2. Oxygen Transport Resistance

Limiting current technique explained elsewhere [38] was employed to determine the oxygen transport resistance (*R*_*O*_2_,*T*_, s/cm) via Equation (5) below.

(5) $$R_{O_{2},T}=\frac{4FC_{O2,ch}}{i_{lim}}=[R_{O_{2},GDL}+R_{O_{2},MPL}^{M}]+[R_{O_{2},MPL}^{Kn}+R_{O_{2},CL}]$$

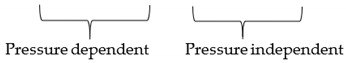

where $C_{O2,ch}$ is gas concentration at the channel, $i_{lim}$ is limiting current density, *F* is Faraday constant. $R_{O_{2},T}$ is a combination of the pressure-dependent and -independent terms. In gas diffusion layer (GDL), pore size is in order of 1–10 μm [39] in microporous layer (MPL) pore size varies from ~50 nm to 1 μm [39,40], and in catalyst layer pore sizes are below 100 nm [41]. Therefore, oxygen transport through the gas diffusion layer and some of the pores of MPL occurs via molecular diffusion and the associated transport resistances are denoted as $R_{O_{2},GDL}$ and $R_{O_{2},MPL}^{M}$, respectively. Transport through smaller pores in MPL occurs via pressure-independent Knudsen diffusion. The associated oxygen transport resistance is denoted as $R_{O_{2},MPL}^{Kn}$. In addition to the GDL and MPL, catalyst layer offers additional pressure-independent resistance $R_{O_{2},CL}$, which includes Knudsen oxygen diffusion resistance as well as oxygen permeation resistance through the thin ionomer film coating of the Pt particles.

**Figure 4:**
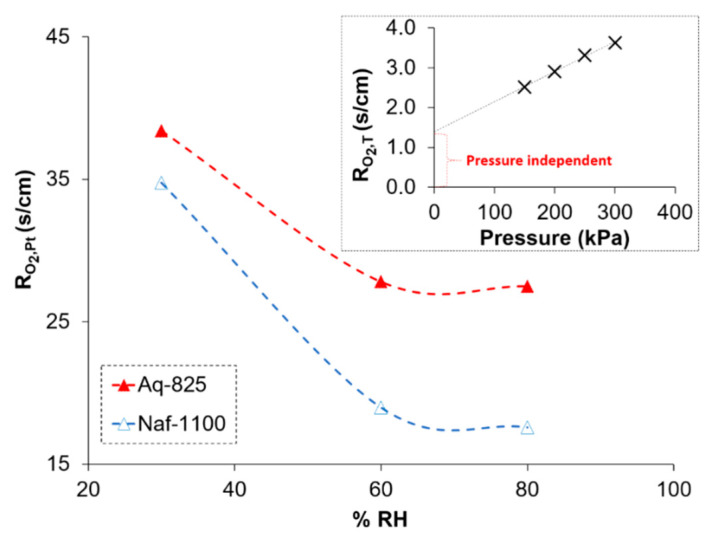
RH-dependent local ionomer transport resistance for both Naf-1100 and Aq-825 catalyst layers estimated at 70 °C. Inset shows total transport resistance as a function of pressure.

The inset in Figure 4 shows an example of the linear increase in $R_{O_{2},T}$ with the pressure. The slope of this line is inversely proportional to the molecular diffusion resistance in GDL and MPL, the pressure-dependent terms, and intercept represents the pressure-independent terms. From our internal study, we estimated $R_{O_{2},MPL}^{Kn}$ is 0.1 s/cm, which is very small compared to the intercept of $R_{O_{2},T}$ ~ 1.5 s/cm. The catalyst layer thickness is determined to be in the 10–12 µm range. Considering the Vulcan carbon support, most of the Pt catalysts are expected to be on the surface of the carbon particle. Thus, Knudsen diffusion within the micropores of carbon support as expected for high surface area carbon can be neglected for the present study wherein Vulcan carbon support has been used. A mix of Knudsen and molecular diffusion through the macro-pores of catalyst layer is expected. In our analyses, the gas phase O_2_ transport resistance is considered to be significantly smaller than local transport resistance. Hence, the magnitude of intercept in the inset mainly corresponds to the $R_{O_{2},CL}$. Accordingly, the average local transport resistance to Pt/ionomer interface ($R_{O_{2},Pt}$) can be approximated via Equation (6) as [9]:

(6) $$R_{O_{2},Pt}=R_{O_{2},CL}\cdot RF$$

$R_{O_{2},Pt}$ in both catalyst layers decrease significantly with RH. Between 30% and 80% RH, Aq-825 exhibits 30% reduction in $R_{O_{2},Pt}$ whereas Naf-1100 shows 50% reduction in $R_{O_{2},Pt}$. The magnitude of $R_{O_{2},Pt}$ for Naf-1100 catalyst layer is similar to the reported values in the literature [9,22]. At each RH, $R_{O_{2},Pt}$ for Naf-1100 catalyst layer is lower than that for Aq-825 catalyst layer, e.g., at 80% RH $R_{O_{2},Pt}$ of Aq-825 catalyst layer is close to 40% higher than $R_{O_{2},Pt}$ for Naf-1100 catalyst layer. Ono et al. also reported higher local $R_{O_{2},Pt}$ for higher IEC ionomers than lower IEC ionomers [31]. RH dependency of $R_{O_{2},Pt}$follows the trend reported by the Toyota group in a study that is the only known direct measurement of oxygen transport resistance of ionomer on Pt [22]. Hydrated ionomer promotes oxygen transport through the ionomer, while the side-chain interactions with Pt likely influences the ionomer thin film morphology, especially the interfacial structure.

## 3. Discussion

In a majority of the prior studies, a key stated motivation of preparing fuel cell catalyst layers with higher IEC ionomer such as Aq-825 is to achieve higher protonic conductance within the catalyst layer (see e.g., Park et al. [29]). However, interfacial processes, whether ORR kinetics or local $R_{O_{2},Pt}$, can also be affected by the ionomer. The higher conductivity of catalyst layer prepared with high IEC ionomer (Aq-825) than that of catalyst layer prepared with low IEC ionomer (Naf-1100) is evident in our study (Figure 2b) and other works [31]. Additionally, our work indicates that the ionomer in a catalyst layer also affects the ORR electrochemical kinetics, an aspect previously not investigated in depth in other studies. Consistent with previous findings, a significant influence of CL ionomer on microstructure-dependent properties (i) Pt utilization (Figure 2a) and (ii) local oxygen transport (Figure 4) is also noted. Our study elucidates the RH dependency of these properties, which has been investigated to a limited extent.

**Figure 5:**
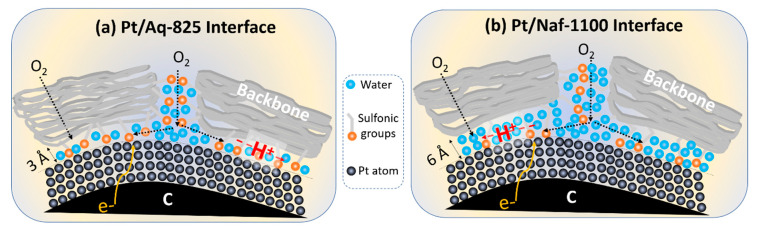
Depiction of Pt/ionomer interface based on neutron reflectometry (NR) at 97% RH (**a**) Pt/Aq-825 interface (**b**) Pt/Naf-1100 interface.

A key point we would like to emphasize is that the interfacial properties are significantly affected by the nature of Pt-ionomer interface. In a previous neutron reflectometry (NR) study from our group [12,14], we examined the temperature- and RH-dependent bulk and interfacial water distribution in 15 nm ionomer films on planar Pt substrates for different ionomers including 3M EW-725 (SSC) and Nafion-1100 (LSC). Based on these findings, Figure 5 depicts the Pt/Aq-825 and Pt/Naf-1100 interfaces [12,14,42]. Although 3M-725 is a different ionomer from Aq-825, both of them have same backbone and similar side chain length, the only difference is spacing between the ionic groups. At 97% RH, Pt/Aq-825 interface may have only a monolayer of water (3 Å) separating Pt surface from the polytetrafluoroethylene (PTFE) backbone. In same condition, two monolayers thick water (6 Å) is formed at Pt/ionomer interface. In both cases, sulfonic groups are shown to interact with Pt surface (based on IR [43]), and hydrophobic back is separated by hydrophilic domains (based on GISAXS [15]).

### 3.1. Pt Utilization

At each RH investigated in our study, Aq-825 catalyst layer exhibited 1.4 times higher ECSA than Naf-1100 catalyst layer. Pt utilization is essentially a quantification of accessibility of protons to the Pt catalysts. Ionomer coverage and poisoning effect are two factors that can affect the Pt utilization.

Naf-1100 has a longer side chain with two ether groups, while Aquivion-825 has a shorter side chain with only one ether group. The combination of RDE and surface-enhanced infrared absorption spectroscopy provides evidence of absorption of the oxygen atom of flexible LSC ether group on Pt atom. Such absorption of oxygen atom of SSC ether group on Pt atom is absent. Thus, a higher blockage of Pt sites is observed in catalyst layer made with LSC ionomer (Nafion) compared to that made with SSC ionomer (Aq-825), resulting in a lower ECSA for Naf-1100 catalyst layer [7]. In absence of any microstructural characterization, we cannot ascertain to what extent ionomer coverage differences contribute to the Pt utilization differences.

### 3.2. ORR Kinetics

As discussed earlier, the differences in the Pt-area normalized kinetic current for the two catalyst layers at a given overpotential is attributed to the interfacial proton concentration. As discussed above, the interfacial water and the sulfonic acid abundance at the interface both will affect the interfacial proton concentration. At low RH (30% RH), the ORR kinetic currents for Aq-825 and Naf-1100 catalyst layers are similar. At this RH, the interfacial water content is expected to be similar for both CLs. It would then appear that there is very little difference in sulfonic acid abundance at the Pt/ionomer interface for the two CLs. At higher RH, significant differences are observed. As depicted in Figure 4 above, there will be higher amount of water at the Pt/ionomer interface in the Naf-1100 CLs. This would be tantamount to diluting the sulfonic acid by different amounts of water, effectively lowering the interfacial proton concentration. Using Equation (7) below, ratio of proton concentration is estimated to be ~3, and the kinetic current of Aq-825 is nearly 4 times greater than Naf-1100.

(7) $$C_{H^{+},interface}\;\propto \;\frac{IEC}{\phi_{w,interface}}$$

where $C_{H^{+},interface}$ is the interfacial proton concentration and $\phi_{w,interface}$ is the interfacial water volume fraction.

### 3.3. Oxygen Transport Resistance

The oxygen transport to the Pt in a catalyst layer comprises of gas-phase transport (mix of Knudsen and molecular diffusion depending on the local pore dimension), transfer from gas-phase to the ionomer phase, diffusion through the ionomer film, and then additional interfacial resistance. For high surface area carbon and agglomerated Pt/C structure, diffusion through the micropores within carbon and pores inside the agglomerate structure, respectively, would also have to be considered. At low Pt loadings, ionomer thin films in the catalyst layer thought to be large contributors to the local oxygen transport resistance [9,11]. The local ionomer thin film resistance for both catalyst layers significantly decreases with RH similar to reported by an ex-situ oxygen transport study on Nafion thin film on Pt [22]. Within the ionomer thin films, oxygen has two transport pathways: (a) through the free volume within the hydrophobic matrix, and (b) via the water-filled hydrophilic domains. Oxygen has relatively high solubility in the hydrophobic Teflon-like matrix but restrictive diffusion. On the other hand, oxygen can be solvated in the water phase of hydrophilic domains and be diffused with ease. As RH increases, both the bulk and interfacial water content increases, thereby making the oxygen transport more facile [11,22]. At same RH, water volume fraction in bulk of Aq-825 ionomer film is expected to be higher than that Naf-1100 ionomer film. However, as depicted in Figure 5, the interfacial water layer thickness for Naf-1100 is almost two times the thickness of Aq-825 interfacial layer. The local oxygen transport resistance ($R_{O_{2},Pt}$) at 80% RH for the Aq-825 catalyst layer is 1.6 times higher than that for the Naf-1100 catalyst layer. If the oxygen transport through the bulk portion of the ionomer film rather than near interface region was the dominant resistance, Aq-825 catalyst layer would not exhibit higher R_O2_ than Naf-1100 catalyst layer. It is hypothesized that the higher interfacial water content at the Pt/ionomer interface in the Naf-1100 catalyst layer promotes faster oxygen transport than that in the Pt/Aq-825 catalyst layer with low interfacial water content.

## 4. Materials and Methods

### 4.1. Catalyst Ink Preparation

The catalyst inks were prepared using commercially available 10.2 wt% Pt/C (Tanaka) electrocatalyst and Nafion (EW 1100) purchased from Ion power Inc. (New Castle, DE, USA) and Aquivion (EW 825) purchased from Sigma Aldrich (St. Louis, MO, USA). The ionomer to carbon (I/C) and solid to liquid (S/L) ratio was maintained at 0.8 and 0.1, respectively. The ionomer stock dispersion was first diluted using a mixture of deionized (DI) water and isopropanol, and the resulting mixture was probe-sonicated for 2 min using an ice jacket to break up the ionomer aggregates. Then, 285 mg of Pt/C catalyst was added into the diluted ionomer dispersion. Then, the mixture was bath sonicated for 15 min followed by three hours of magnetic stirring and 24 h of ball milling.

### 4.2. Membrane Electrode Assembly (MEA) Preparation

The catalyst layer decals were prepared by coating the catalyst inks on ethylene tetrafluoroethylene (ETFE) sheet using a micrometer adjustable film casting doctor blade (EQ-Se-KTQ-250, MTI corporation, Richmond, CA, USA) and air dried for 24 h. The doctor blade was adjusted to a thickness of 100 μm for coating of both catalyst layer decals. Decal transfer method was used to prepare an MEA. Nafion membrane (25 μm, NRE-211, DuPont, Wilmington, DE, USA) was hot-pressed in between the anode (0.2 mg_Pt_/cm^2^) and the cathode decals at 150 °C and 2 MPa pressure for 3 min [44,45]. The MEAs had an anode and cathode active area of 1.44 and 1 cm^2^, respectively. The larger anode area was maintained to ensure redundant supply of reactant (protons) from the anode. The thickness, platinum loading, and ionomer loading of the cathode CLs are listed in Table 1. The changes in local and interfacial transport properties become very insignificant and remain obscured at high cathode Pt loading as it scales inversely with Pt ECSA, thus CLs with ultra-low Pt loading were prepared in this study.

### 4.3. Fuel Cell Assembly and Testing

A small scale and differential flow field cell was assembled by sandwiching the hot-pressed MEA between 160 µm thick Toray gas diffusion layers coated with microporous layer (MPL) (TGP H-60, Toray, Japan). The differential cell was used to avoid any discrepancies arising from the gradient in RH and reactant gas concentrations along the active area. The single cell was tested using a fuel cell test station (100W, Greenlight Innovation, Burnaby, Canada) coupled with two potentiostats (Biologic SP-200, Seyssinet-pariset, France and Ivium Vertex, Eindhoven, The Netherlands).

Electrochemical impedance spectroscopy (EIS), cyclic voltammetry (CV), and linear sweep voltammetry (LSV) tests were performed under H_2_/N_2_ for determining high-frequency resistance (HFR) for the electrodes, ECSA, and H_2_ crossover, respectively. The CV and LSV tests were performed at 200 and 5 mV/s, respectively. The protonic resistance of ionomer in the cathode CL was determined by performing EIS at 0.4 V under H_2_/N_2_ by sweeping frequencies in the range of 1 Hz to 1 MHz with an amplitude of 10 mV. Pt electrochemical surface area (ECSA) was determined by integrating the hydrogen underpotential deposition (H_upd_,_desorption_) region while subtracting capacitive currents. The cell was initially conditioned (see Table 2 for the conditioning protocol details).

The cell performance was characterized by collecting potentiostatic polarization curves with in-situ high-frequency resistance (HFR) using an Ivium Vertex potentiostat (Ivium, Eindhoven, Netherlands). The potentiostatic polarization curve was obtained by holding the voltage for 3 min at each voltage at a resolution of 0.1 V. The limiting current study was performed by varying O_2_ concentrations (1–24% O_2_:N_2_) [38,46]. The detail of all the testing conditions is outlined in Table 3.

## 5. Conclusions

In this work, the effect of ionomer side chain length (or EW) on the electrochemical interfacial properties and mass transport properties including long-range proton transport, local O_2_-transport, Pt utilization, double-layer capacitance, and ORR reaction kinetics was investigated at varying RH. In summary, the CL prepared with shorter side chain (Aq-825) exhibited higher ECSA, higher CL ionic conductivity, higher CL double-layer capacitance, and higher CL local O_2_-transport resistance compared to the CL prepared with longer side chain (Naf-1100). The differences in these properties can be explained on the basis of differences in the EW and side chain. However, the differences in catalyst layer microstructure such the ionomer coverage and connectivity or the pore size can also be responsible. A systematic study combining microstructural characterization and catalyst layer properties is needed to ascertain the origin of the observed differences in catalyst layer properties. At 120 mV ORR overpotential, the Aq-825 CL showed 2-5 times higher ORR activity compared to the Naf-1100 CL at any given RH, except at 30% RH, which can be ascribed to the higher interfacial concentration of protons at the Pt/ionomer interface for Aq-825 CL. Our findings also indicate that at each RH, Naf-1100 CL illustrated lower local oxygen transport resistance ($R_{O_{2},Pt}$) compared to Aq-825 CL, for instance, Aq-825 CL showed 40% higher $R_{O_{2},Pt}$ at 80% RH. For both CLs, $R_{O_{2},Pt}$ decreased with increasing RH as higher interfacial water content at the Pt/ionomer interface in the CL promotes faster oxygen transport.

## Figures and Tables

**Table 1:** Summary of the cathode catalyst layer properties.

Catalyst Layer Type	CL Thickness (µm)	Pt Loading (mg_Pt_/cm^2^_geo_)	Ionomer Loading (wt%)	Ionomer Loading (mg/cm^2^_geo_)
Nafion	11 ± 1	0.043	42.07	0.34
Aquivion	10 ± 1	0.036	42.04	0.28

**Table 2:** Summary of the conditioning protocol used in this study.

Step #	Test	T_cell_ (°C)	T_hum_ (°C)	Flow Rate An/Ca (NLPM)	Reactant Gas An/Ca	Back Pressure (kPag)	Time (hrs)
1	H_2_ pumping	30	45	0.5/0.5	H_2_/H_2_	0	0.5
2	Flooding	60	70	0.05/0.1	H_2_/N_2_	50	8–12
3	0.6 V hold	70	70	0.3/0.5	H_2_/O_2_	200	12
4	Potential cycling (0.6–0.8 V)	70	70	0.3/0.5	H_2_/O_2_	50	Holding 5 min at each potential

**Table 3:** Summary of membrane electrode assembly (MEA) testing conditions for all the tests performed in this study.

Test	T_cell_ (°C)	RH (%)	Flow Rate An/Ca (NLPM)	Reactant Gas An/Ca	Back Pressure (kPag)
Diagnostics (CV, LSV, EIS)	70	30, 45, 60, and 100	0.1/0.2	H_2_/N_2_	50
Performance			0.3/0.5	H_2_/Air	
Limiting current		30, 60, and 80	0.2/0.5	H_2_/O_2_ (1–24%):N_2_	50, 100, 150, and 200
